# Supplementary material for: Are carbon emissions trading and green financial instruments synergistic? -Comprehensive quantitative research based on content analysis
Source: PLoS One. 2024 Mar 7;19(3):e0298601. doi: 10.1371/journal.pone.0298601 (PMC10919601; doi:10.1371/journal.pone.0298601)
Supplement: S1 Data — (ZIP) [file pone.0298601.s001.zip › Data/地级市Z_F工作报告.zip!/╒π╜¡/║■╓▌╩╨/└┤╫╘╣1⁄2╓┌║┼í╛┬φ┐╦╩2╛▌═°í┐_2016.docx]

各位代表：

现在，我代表市人民政府，向大会作工作报告，请予审议，并请市政协委员和其他列席人员提出意见。

一、“十二五”发展和2015年工作回顾

“十二五”时期，是湖州发展砥砺奋进、很不平凡的五年。五年来，全市人民在中共湖州市委的领导下，紧紧围绕打造特色产业集聚区、城乡统筹先行区、生态文明示范区、幸福民生和谐区，建设现代化生态型滨湖大城市的奋斗目标，积极应对国际金融危机持续影响等一系列重大风险和挑战，顽强拼搏，开拓进取，全市经济社会发展取得新的成就。“十二五”规划《纲要》确定的主要目标任务顺利实现，湖州在全面建成小康社会的征程上迈出了坚实有力的步伐。

——产业提升取得新的突破。地区生产总值从1301.7亿元增长到2084.3亿元，年均增长9.2%，比全省快1个百分点，按常住人口计算，人均达到70899元。三次产业结构从8∶54.9∶37.1调整到5.9∶49.2∶44.9。工业“3＋3”产业增加值占比达到53.3%，长兴、德清相继跨入规上工业产值超千亿县行列。服务业加速扩张，休闲旅游等重点产业和电子商务等新兴业态蓬勃发展。农业现代化发展指数列全省第一位。财政总收入从172.3亿元增加到327.8亿元，其中地方财政收入从97.3亿元增加到191.3亿元，按可比口径计算，年均分别增长13%和13.2%，比全省快1.9和1.6个百分点。

——城乡建设呈现新的面貌。城市化率从58%提高到62.2%。基础设施水平大幅提升，湖州迈入“高铁时代”，县区全部通上高速公路。月亮酒店、东吴国际广场等一批重大地标性建筑相继建成，新区展现活力，旧城焕发新貌，城市功能和品质持续提升。我市成为国家历史文化名城。市级美丽乡村建成率超过70%，省级美丽乡村创建先进县区实现全覆盖。

——生态文明迈向新的阶段。“绿水青山就是金山银山”的发展理念深入人心，“生态＋”的发展方式加快形成。生态环境保护和治理力度不断加大，节能减排各项约束性指标全面完成，水环境和空气质量稳步改善。我市成为国家森林城市，在全国地级市中成为首个生态文明先行示范区，有望在全省率先实现国家级生态县区全覆盖，有望成为全省首个国家生态市。安吉县荣膺“联合国人居奖”。

——民生和谐达到新的水平。城镇居民、农村居民人均可支配收入分别从25344元、14143元增长到42238元、24410元，年均增长10.8%和11.6%。家庭人均年收入低于4600元的贫困现象全面消除。统筹城乡的社会保障体系从制度全覆盖走向人群全覆盖，保障水平逐年提高。基本公共服务均等化程度不断提升，教育、文化、卫生、体育等各项社会事业协调发展。法治湖州和平安湖州建设相互促进，社会保持和谐稳定。

——改革开放增添新的活力。政府自身改革不断深化，城乡体制改革深入推进，资源要素市场化配置改革全面提速。融入经济全球化和区域一体化的进程明显加快，投资和贸易领域“引进来”、“走出去”的规模不断扩大。国家创新型试点城市建设取得积极进展，全社会研究与试验发展经费支出相当于地区生产总值的比例从1.64%提高到2.5%以上，每万人有效发明专利拥有量达到12.9件。

这些成就的取得，标志着湖州改革开放和现代化建设事业又迈上了一个新的台阶，极大增强了全市人民的自信心和自豪感，必将激励我们继续奋勇前进。

刚刚过去的一年，我们主动适应经济发展新常态，全力打好转型升级组合拳，统筹推进民生改善和社会建设，较好地完成了市七届人大五次会议确定的目标任务和十方面民生实事项目。地区生产总值增长8.3%，增速列全省第三位；财政总收入和地方财政收入分别增长7.1%和7.3%；城镇居民和农村居民人均可支配收入分别增长8.4%和9%。一年来，我们重点做了以下工作：

（一）统筹稳增长调结构，经济发展稳中有进、转中向好

有效投资持续扩大。开展季亮项目活动，全力打好项目建设攻坚战，完成固定资产投资1402.6亿元，增长12.9%。加快重大基础设施建设，四大骨干水利工程全面推进，商合杭铁路湖州段、申嘉湖高速公路西延、长龙山抽水蓄能电站等项目动工兴建。聚焦“大好高”项目引进推进，全力谋划大产业、招引大项目，新引进首期固定资产投资10亿元以上项目25个，签约百亿级项目5个。大力提升平台发展能级，全市新拓展平台18.9平方公里，省级吴兴经济开发区获批，湖州丝绸小镇、南浔善琏湖笔小镇、德清地理信息小镇入选全省首批特色小镇创建名单。

产业升级切实加快。全力推进工业转型升级，规上工业增加值增长6.4%，增速列全省第二位，其中战略性新兴产业、高新技术产业、装备制造业分别增长9%、9.8%和7.8%，高新技术产业对规上工业的贡献率达到61.3%。长兴县被列为全省唯一的工业转型升级示范区试点。统筹“四换三名”和“两化”深度融合，淘汰239家企业的落后产能，完成工业技改投资443.4亿元。大力提升服务业发展水平，第三产业增加值增长11.9%，增速列全省第三位。完成社会消费品零售总额963.9亿元、网络零售额223.6亿元，分别增长10.6%和84%。加快内河水运和临港经济转型发展，内河集装箱吞吐量达到18.5万标箱，增长52.7%。实施“12＋8”政策措施，房地产市场保持平稳健康发展，商品房销售面积增长30.9%。累计建成粮食生产功能区超过70万亩，全面建成11个省级现代农业综合区。现代生态循环农业试点市建设扎实推进。吴兴区在国家现代农业示范区农业改革与建设试点绩效评价中获得第一名。

创新成果不断显现。我市获批国家科技创新服务体系建设试点。湖州莫干山高新区跨入国家级行列，长兴国家大学科技园建成开园，创业园、微总部、创新工场等众创空间蓬勃兴起。全市新引进创新载体31家，第六届政产学研合作大会签约项目73个。工业企业质量标准品牌升级行动扎实推进，新增“质量强、标准强、品牌强”企业60家。建立紧缺急需人才引进校地战略合作联盟，新入选“国千”人才7名、“省千”人才21名、省领军型创新团队3个，“南太湖精英计划”新引进领军型创业创新团队166个。全面实施“金象金牛”大企业、“高技术、高成长”企业培育和小微企业成长三个计划，新增上市公司7家、新三板挂牌企业26家，新增国家重点扶持高新技术企业73家，完成“个转企”1335家、“小升规”254家、“下转上”258家。

（二）深化改革开放，市场化发展活力持续增强

政府自身改革深入推进。完善“四张清单一张网”，开通政务服务网上审批功能，建立行政审批网上中介超市。实施“五证合一、一照一码”企业注册登记制度改革，新增市场主体3.6万户。完成新一轮政府机构改革，新组建市卫生计生局，单设市规划局，推进综合行政执法体制改革，建立并实施不动产统一登记制度。推进财政预算制度改革，加强政府性债务管理，财政资金使用监管机制进一步完善。

要素配置改革切实加快。加强建设用地节约集约利用综合评价，在湖州开发区开展以“破僵尸企业、破围墙圈地、破低效用地、破既得利益”专项整治为重点的改革试点，全市新增建设用地指标25839亩，盘活存量建设用地8062亩，复垦利用废弃工矿地7796亩。推进金融业改革，加快金融业发展，组织“百名行长进千企”大走访，金融机构贷款余额新增172亿元，银行业不良贷款率持续低于全省水平，完成直接融资445亿元，新增各类金融新业态企业440家。深化投融资体制改革，建立政府与社会资本合作项目库，设立政府性产业投资引导基金。

开放型经济发展水平不断提升。全力抓好招商引资，浙商回归省外到位资金239.3亿元，实到外资9.4亿美元。推进外贸创新驱动拓市场试点，建成全市首家外贸综合服务平台，安吉上港园区入选省级跨境电子商务园区。进出口总额达到636.4亿元，增长3.7%，增速列全省第三位，其中出口552.5亿元，增长2.1%。服务贸易、境外投资等迅速发展。进一步加强对口支援和对外合作，新增国外友城3个，与北京市西城区建立友好区市关系。

（三）加快城乡一体化进程，新型城市化扎实推进

中心城市建设不断迈上新台阶。一体化推进南太湖滨湖区域规划建设，组团式推进湖州开发区西南分区、吴兴东部新区、南浔城区建设发展。中心城市外环道路全线基本建成，怡和家园、仁北小学全面竣工，市民服务中心、奥体中心主体工程顺利结顶，太湖水厂、五一大桥等项目加快建设。各县区一批重点城建项目扎实有序推进。“三改一拆”超额完成年度目标，交通治堵不断深化，城市精细化管理进一步加强。

美丽乡村建设进一步提档升级。由安吉县主导制订的美丽乡村建设指南成为国家标准，全市累计基本建成19条美丽乡村示范带。德清县在首次农村人居环境村村普查中得分全国最高。开展永久基本农田划定工作，提高垦造耕地奖励补助标准。我市农业科研与技术推广体制创新改革试验成果在全省推广。完成农村公路等级提升224.5公里，新增农村生活污水治理受益农户7.7万户，农村垃圾减量化、资源化利用和推广试点扎实推进，农业电力线路改造进一步加快。完成座家船清理拆解工作。

城乡体制改革向纵深推进。深入推进农村产权制度改革，“三权”确权进一步深化，各县区农村综合产权流转交易平台全面建成，全省首宗农村集体经营性建设用地拍卖入市在德清县顺利完成。我市被列入全国中小城市综合改革试点。小城市培育和中心镇改革发展继续深化。“多规合一”试点稳步实施。在全省率先完成户籍制度改革，原来依附在户口性质上的城乡公共政策差别明显缩小。

（四）打造生态环境生产力，生态文明建设走在前列

生态环境治理进一步深化。大力度推进“五水共治”，在全省率先消灭市控断面劣V类和V类水，三县全部创建为省级“清三河”达标县。持续开展治霾“318”攻坚行动，淘汰高污染燃料小锅炉2942台、黄标车10874辆，实施电能替代项目172个，市区PM2.5日均浓度每立方米56.9微克，下降11.4%。实施新一轮“四边三化”行动，“两路两侧”环境治理有效推进。完成造林更新1.9万亩、平原绿化2.9万亩。在全国率先开展绿色矿业发展示范区建设，矿山开采减点控量和打非治违继续保持高压态势。

生态文明制度和生态文化建设加快推进。围绕“绿水青山就是金山银山”重要思想提出十周年，举办一系列纪念和研讨活动，湖州生态文明建设的影响力进一步扩大。编制完成环境功能区划，率先开展国家生态文明标准化示范区建设，我市被列入自然资源资产负债表编制和领导干部自然资源资产离任审计国家试点，市级环境权益交易中心挂牌运行。成立中国生态文明研究院，开建浙江省自然博物园。推行《市民生态文明公约》，设立“湖州生态文明日”，生态文明建设的公众认同感、参与度不断提升。

生态经济蓬勃发展。出台“生态＋”行动实施意见，努力打造绿色发展高地。大力提升休闲旅游业发展水平，我市列入首批国家级旅游业改革创新先行区，国际乡村旅游大会永久会址落户湖州，成功创建国家级太湖旅游度假区和省级西塞山旅游度假区，南浔古镇成为全市首个国家5A级景区，安吉欢乐风暴乐园等一批项目建成运营，长兴太湖龙之梦乐园等一批项目成功签约，全市旅游总收入达到700亿元，增长39.1%。谋划和推动健康产业加快发展，鑫远?太湖国际健康城等一批项目开工建设。新能源汽车等新兴绿色产业快速发展，长兴县成为全省首个纯电动汽车及关键零部件产业发展基地。

（五）加强社会建设，民生持续改善、社会和谐稳定

就业促进和社会保障进一步加强。支持大众创业，促进充分就业，城镇新增就业7.6万人，登记失业率2.67%。深入构建和谐劳动关系，企业建立工资集体协商制度的覆盖率稳步提高。完成全民参保登记工作，实施机关事业单位养老保险制度改革，社会保障持续扩面提标。推行廉租房和公租房制度并轨，在全国率先开展住房公积金贷款资产证券化试点。整合社会救助资源，完善医疗救助和临时救助制度，完成城乡最低生活保障同标提标。慈善、红十字事业健康发展，残疾人权益有效保障。以居家为基础、社区为依托、机构为支撑的养老服务体系加快建设。

社会事业发展水平不断提升。深化教育教学改革，促进各类教育优质均衡发展，一类重点大学上线率首次突破15%。德清成为全省首批基本实现教育现代化县，我市被列为全国首批现代学徒制试点城市和国家特殊教育改革实验区。高等教育发展水平持续提升。上海交通大学教育集团湖州现代教育示范园区开工建设。推进公立医院综合改革，实施“双下沉、两提升”工程，增强基层医疗卫生服务能力，公共卫生综合发展指数位居全省第一。联合国人口基金“第七周期”项目圆满完成。推动社会力量参与公共文化服务，组建“城市文化联盟”。新建成农村文化礼堂90个，农村应急广播体系实现行政村全覆盖。钱山漾文化遗址被命名为“世界丝绸之源”。成功举办市第八届运动会等重大赛事。

平安湖州建设扎实推进。全面深化法治湖州建设，顺利完成“六五”普法，实施阳光执法司法工程，公共法律服务体系建设加快推进。加强立体化社会治安防控体系建设，完善矛盾纠纷排查化解机制，全面完成社会管理重点项目建设三年行动纲要，群众对平安创建工作满意度列全省第一。严格落实安全生产主体责任、监管责任，开展重点行业领域事故隐患排查整治等专项行动，各项控制性指标连续十年实现负增长。加强餐桌安全治理，推进食品安全基层责任网络规范化建设。新居民管理服务基础有效夯实。社会应急联动体系不断完善。深化国防动员和全民国防教育，推进军民融合深度发展，顺利通过全国双拥模范城“七连冠”检查验收。民族、宗教、侨务、对台等工作进一步加强。

（六）坚持依法行政、为民勤政，法治政府和服务型政府建设进一步加强

法治政府建设加快推进。出台深入推进依法行政加快建设法治政府实施办法，完善政府履行职能的清单管理制度。健全政府法律顾问和常务会议学法制度，完善行政决策程序，加强行政执法规范化建设，推行行政处罚结果和行政复议决定网上公开。自觉接受人大、政协等各方面的监督， 220件市人大代表建议和300件市政协提案顺利办结。

作风建设和廉政建设切实加强。认真开展“三严三实”专题教育，持之以恒正风肃纪。组织服务企业系列活动，落实清费减负政策。“阳光湖州服务指数”逐季提升，“12345政府阳光热线”被评为第三届中国民生发展论坛“2015民生示范工程”第一名。全面推行政府系统履行党风廉政建设主体责任报告制度，加强重点部门、重点领域廉政风险防控，健全和完善权力运行制约和监督体系。专项治理损害群众利益行为，严肃查处违纪违法案件。

各位代表！“十二五”规划胜利收官，成绩来之不易。这是省委、省政府和市委科学决策、正确领导的结果，是全市干部群众齐心协力、攻坚克难的结果。在此，我代表市人民政府，向全市人民和外来建设者，向全体人大代表、政协委员、离退休老同志，向各民主党派、工商联、人民团体和社会各界人士，向驻湖人民解放军和武警部队官兵，向所有关心和支持湖州发展的海内外朋友，表示衷心的感谢，致以崇高的敬意！

我们既要看到成绩，更要看到前进中的困难和挑战。受宏观环境等因素影响，“十二五”期间地区生产总值以及2015年财政收入、外贸出口等未能达到预期目标。特别是经济企稳向好的基础还不稳固，部分行业产能过剩问题比较突出，企业生产经营困难增多；创新能力、投资效率等有待提升，新增长点的支撑效应还不够；群众对医疗、养老、教育、公共安全等还有一些不满意的地方，水环境和大气治理任务仍然艰巨；政府转变职能、提升效能还有差距，一些领域消极腐败现象时有发生。我们一定要保持清醒头脑，坚定信心、下定决心，直面问题、勇于担当，努力把各项工作做得更好。

二、“十三五”时期经济社会发展主要目标和重点任务

根据《中共湖州市委关于制定湖州市国民经济和社会发展第十三个五年规划的建议》，我们在广泛听取意见、反复研究论证的基础上，编制了《湖州市国民经济和社会发展第十三个五年规划纲要（草案）》，提请大会审议。

“十三五”是我国全面建成小康社会的决胜阶段，也是湖州在经济发展新常态下突破瓶颈制约、实现动力转换的攻坚时期。世界经济有望在深度调整中曲折复苏，但不稳定不确定因素依然较多。国内经济长期向好基本面没有改变，但发展不平衡、不协调、不可持续问题仍然突出。区域一体化发展进程明显加快，但城市间围绕创新资源、高端要素展开的竞争将更加激烈。我市区位优势明显、生态禀赋独特、发展潜力巨大，但综合实力不强、发展方式粗放的矛盾依然存在，赶超发展面临既要扩量又要提质的双重艰巨任务。综合判断，我们正处在一个激流勇进、不进则退的关键历史节点，必须以坐不住、等不起、慢不得的责任感和紧迫感，奋发有为抓发展，趋利避害求突破。

“十三五”时期我市经济社会发展的指导思想是：

高举中国特色社会主义伟大旗帜，以马克思列宁主义、毛泽东思想、邓小平理论、“三个代表”重要思想、科学发展观为指导，深入贯彻习近平总书记系列重要讲话精神，以“四个全面”战略布局为统领，坚定不移照着“绿水青山就是金山银山”这条路走下去，坚定不移以“八八战略”为总纲打好转型升级系列组合拳，坚定不移坚持一张蓝图绘到底，坚持以创新、协调、绿色、开放、共享的发展理念引领赶超发展，实施“生态立市、工业强市、产业兴市、开放活市”战略，扎实推进重大项目、重点工程、重大平台、重点产业、重大政策、重点要素工作，统筹推进经济建设、政治建设、文化建设、社会建设、生态文明建设和党的建设，全力打造创新创业先行区、特色产业集聚区、城乡统筹样板区、生态文明示范区、幸福民生和谐区，加快建设现代化生态型滨湖大城市，高水平全面建成小康社会。

“十三五”时期，围绕高水平全面建成小康社会，我们要努力实现“六个更加”的目标：

——经济实力更加壮大。地区生产总值年均增长8%以上，到2020年，人均生产总值力争达到或超过全省平均水平。财政总收入、地方财政收入年均增长7.5%左右。战略性新兴产业、高新技术产业占比持续上升，努力培育形成四大千亿级重点主导产业集群。服务业比重上升到50%，形成“三二一”产业格局。农业现代化发展水平继续保持领先。

——发展活力更加激发。全社会研究与试验发展经费支出相当于地区生产总值的比例提高到2.85%，发明专利授权量年均增长10%以上，新增各类人才20万名，努力进入创新型城市行列。“引进来”和“走出去”齐头并进，开放发展水平全面提升。

——城乡区域更加协调。市域空间发展格局进一步优化，城市化率提高到68%。中心城市集聚和辐射能力显著增强，市本级持续快速发展，县域经济加快向城市经济转型，小城市、中心镇培育成效明显，美丽乡村全面打造升级版。

——生态环境更加优美。确保完成节能减排各项约束性指标，市控以上监测断面Ⅰ-Ⅲ类水质比例提高到95%以上， PM2.5浓度降幅超过全省平均水平。全国生态文明先行示范区建设走在前列，生态环境生产力大幅度提升，形成可复制、可推广的“湖州模式”。

——人民生活更加幸福。居民人均可支配收入年均增长8%以上，努力实现全体人民共同迈入全面小康。教育、文化、卫生、就业创业、社会保障等基本公共服务均等化水平稳步提升。居民思想道德素质、科学文化素质、健康素质明显提高，努力创建成为全国文明城市。

——治理体系更加完善。市场在资源配置中起决定性作用和更好发挥政府作用得到全面落实，城乡发展一体化、要素配置市场化、生态经济融合化、社会治理法治化等重大体制机制改革向纵深推进，法治政府基本建成，群众安全感满意率保持在全省前列。

为实现上述目标，《纲要（草案）》提出了“十三五”时期我市经济社会发展的任务。我们要以五大发展理念为引领，以“六重”为抓手，着力抓好以下工作，加快推动赶超发展：

（一）突出制度供给，强化创新驱动。深化行政管理体制改革，持续推进简政放权、放管结合、优化服务，进一步转变职能、提高效能，激发市场活力和社会创造力。强化“非禁即入、亩产效益”导向，完善宽进严管的审批监管制度、集聚集约的要素配置制度、扶优助强的服务保障制度。以推进国家创新型试点城市和国家知识产权示范城市建设等为抓手，深化科技体制改革，完善以企业为主体、市场为导向、政产学研金深度合作的区域创新体系，促进更多科技成果更好产业化。全面加强人才队伍建设，加快打造特色小镇，大力发展众创、众包、众扶、众筹等新模式，孵化和培育新经济、新业态。

（二）坚持工业强市，加快产业兴市。顺应产业跨界发展和融合发展新趋势，加快发展信息经济、高端装备、健康产业、休闲旅游四大重点主导产业，改造提升金属新材、绿色家居、现代纺织三大传统优势产业，积极培育新能源汽车、地理信息、美妆时尚、现代金融等若干个引领未来发展的新增长点。对接《中国制造2025》，加快“四换三名”、“两化”深度融合，提升“湖州制造”水平。大力发展现代服务业，推动生产性服务业向专业化和价值链高端延伸、生活性服务业向精细化和高品质转变。以休闲、精致和绿色、循环为导向，加快转变农业发展方式。以省级以上集聚区、开发区、高新区、度假区等为主战场，着力打造主导产业突出、集聚效应明显、产城融合发展的产业高地。

（三）建设生态文明，打造美丽湖州。谋划实施新一轮“811”美丽湖州建设行动，加快经济生态化、生态经济化。全面实施规上企业资源节约型、环境友好型改造，推动再生资源回收利用体系建设，大力发展绿色经济、循环经济、低碳经济。继续铁腕治水治气治土治矿，推进用能用水用地排污等总量和强度“双控”行动，做到不把违法建筑、污泥浊水、环境“脏乱差”带入全面小康。加快生态文明制度创新，基本形成源头预防、过程控制、损害赔偿、责任追究的制度体系。

（四）加强对接合作，提升开放层次。完善和提升高速铁路网、高速公路网、干线公路网、航道水运网，重点构建由杭湖宁、商合杭、湖苏沪铁路组成的“十字”型高铁枢纽发展格局。深入对接“一带一路”、长江经济带，积极参与“网上丝绸之路”建设。深化接轨沪杭、连通苏皖，加强与上海等资本、技术、人才密集型城市的合作交流，主动融入长三角一体化和杭州都市圈建设。把占领国际国内市场更大份额作为开放发展的着力点，谋划设立综合保税区，创新投资贸易发展方式和促进体系。

（五）加快城乡一体，促进均衡协调。启动新一轮城镇体系规划修编，做大做强湖州中心城市发展极核，一体化提升滨湖发展带，特色化提升临杭发展带，培育壮大高速公路、高速铁路沿线经济发展轴，进一步优化城镇空间布局、功能等级、人居环境。更加突出宜居宜业宜游，注重自然风貌保护和文化特色传承，全域化打造美丽乡村升级版。深化城乡体制改革，促进城乡公共资源均衡配置，推动城镇公共服务向农村延伸，维护进城落户农民土地承包权、宅基地使用权、集体收益分配权并支持引导依法自愿有偿转让。

（六）优化共建共享，增进民生福祉。大力支持以创业带动高质量就业，着力解决结构性就业矛盾。实施全民参保计划，完善覆盖城乡居民、更加公平可持续的社会保障制度。深化收入分配制度改革，增强低收入群体自我发展能力，促进城乡居民持续普遍增收。坚持教育优先发展战略，深化教育领域综合改革，加快实现教育现代化。联动推进医疗、医保、医药改革，增强全民体质，打造健康湖州。加强优质生育服务，提高出生人口素质。积极应对人口老龄化，大力发展老龄服务事业和养老服务产业。传承和弘扬湖州特色文化，发展文化产业，增加文化供给，加快建设文化强市。完善社会治理体系，加强防灾减灾能力建设，提高公共安全保障水平，全面打造平安湖州。

三、 2016年工作安排

今年是高水平全面建成小康社会决胜阶段的开局之年，也是本届政府完成任期目标的决战之年。宏观经济环境仍然严峻复杂，改革发展稳定任务更加艰巨繁重。差距最容易的是在逆境中拉开，成就最可贵的是在考验中创造。做好今年政府工作，实现“十三五”良好开局，我们必须把适应新常态、把握新常态、引领新常态作为大逻辑，下好先手棋、打好主动仗，切实做到变中求新、新中求进、进中突破；我们必须坚持以五大发展理念引领赶超发展，崇尚创新、注重协调、倡导绿色、厚植开放、推进共享，努力改造旧动能，加快培育新动力；我们必须紧紧围绕去产能、去库存、去杠杆、降成本、补短板，大力推进供给侧结构性改革，着力提高经济社会发展质量和效益；我们必须充分叠加区位、交通优势和生态、人文特色，合力汇聚更多高端发展要素，不断提升城市竞争力和综合实力；我们必须以扎实开展“四个年”活动为抓手，敢于担当、精准发力，依靠实干指数、优政指数换取发展指数和民生指数。

今年全市经济社会发展的主要预期目标为：地区生产总值增长8%以上，财政总收入、地方财政收入均增长7%左右，城乡居民人均可支配收入分别增长8%以上和8.5%以上，节能减排完成省下达任务。重点做好以下工作：

（一）坚持不懈把改革开放推向纵深

深化行政管理体制改革。以“四张清单一张网”为抓手，扎实推进政府职能转变。推进市县行政审批层级一体化改革，优化企业投资项目高效审批，加快工业企业清单外“零增地”技改项目由审批制向备案承诺制转变，完善事中事后监管制度。深入推进综合行政执法体制改革，建立健全联合执法协调机制。建立事业单位统一登记制度和统一社会信用代码制度。严格规范机构编制管理。完善全口径预算体系，深化部门预算改革，优化公共财政支出结构。

加快投融资体制改革。完善政府与社会资本合作项目库，充实储备一批，启动实施一批，示范运作一批。实施市本级经营性国有资产盘活重组，启动政府性产业投资引导基金实质性运作。加强金融服务实体经济，努力扩大信贷规模、优化信贷结构。促进融资担保体系建设，依法严厉打击恶意逃废债行为，保持较低的银行业不良贷款率。继续大力对接资本市场，力争新增上市和挂牌企业30家，完成直接融资300亿元。坚持加快发展与管控风险并重，促进现代金融产业健康成长，新增各类金融新业态企业300家。

提升开放合作水平。实施对外贸易优质优价战略，确保外贸出口持续增长，努力提高湖州出口占全省出口的份额。大力发展服务贸易。主动对接上海自贸区、杭州中国跨境电子商务综合试验区，推动设立海关特殊监管区，打造一批中外合作产业园。完善口岸协调工作机制，提高外贸便利化水平。创建全国内河水运转型发展示范区，建设湖州铁公水综合物流园，大力发展江河海联运。支持有条件的企业设立境外营销网络、合作开发境外资源。发展境外上市、股权投资、融资租赁等利用外资新方式。深化对外交流合作，继续做好对口支援工作。

（二）全力促进经济稳速提质增效

统筹推进企业培育和升级。开展降低实体经济企业成本行动，落实正税、清费、调价、减负等各项政策，不断优化企业发展环境。深入开展“金象金牛”大企业培育，促进龙头企业兼并关联企业，鼓励企业在并购重组中做大做强。深入实施小微企业三年成长计划，引导和鼓励小微企业发展定制经济和个性服务，培育细分行业领军企业。完成工业企业质量标准品牌升级三年行动计划，深化“知名企业、知名企业家、知名品牌”培育工程，新创建40家以上“质量强、标准强、品牌强”企业。

以补短板为重点加大有效投资力度。坚持“大好高”导向，着力谋划大产业、招引大项目，加快签约一批、落地一批、建成投产一批。突出产业链招商、以商引商，省外浙商回归到位资金260亿元左右，实到外资10亿美元。聚焦重大基础设施、重大产业项目、高新技术产业、绿色生态环保以及工业技术改造，努力扩大有效投资，确保固定资产投资增长11%以上，力争市重点建设项目完成投资646亿元，工业“三百计划”完成投资300亿元，服务业百个重点项目完成投资100亿元以上。

以防风险为重点加强经济运行调节。建立健全常态化政企沟通联系机制，积极稳控和化解涉企金融风险，及时协调解决经济运行中的矛盾和困难。妥善处置“僵尸企业”，加快实现市场出清。完善建设用地指标分配与节约集约用地挂钩制度，加强重大项目用地保障。加大对住房消费的支持力度，推进拆迁安置和住房保障货币化改革，促进房地产市场持续平稳健康发展。置换存量、严控新增、降低成本，积极防范政府性债务风险。

（三）大力推进产业转型升级和创新驱动发展

全力培育战略性新兴产业。在四大重点主导产业和新增长点领域确定一批重大项目和龙头企业，实施定向招商，强化重点培育，力争信息经济、高端装备、健康产业、休闲旅游产业增加值分别增长12%、 8%、 10%和10%左右。推进湖州开发区北斗微电子产业园、德清地理信息小镇建设，谋划建设吴兴美妆小镇、长兴新能源小镇、安吉天使小镇、安吉影视小镇、南浔智能电梯小镇、湖州开发区智能电动汽车小镇、太湖度假区健康·蜜月小镇等。大力培育通用航空、文化创意、物流快递等新兴产业。

加快推动传统产业改造提升。以块状经济为重点，深化工业细分行业转型升级。严格执行能耗和限排标准，加快淘汰和整治高污染高耗能的落后产能。推动制造业智能化发展，引导企业提升设计、制造、工艺、管理水平。推进建筑工程总承包和新型建筑工业化两项试点，加快建筑业转型升级。深入实施“互联网＋”行动计划，推动电子商务在规上企业实现全覆盖。加快商贸业转型发展，培育和发展电商平台，努力实现社会消费品零售总额增长10%、网络零售额增长35%左右。加快农业产业结构调整，继续严控温室龟鳖、生猪养殖规模，提升湖羊、茶叶、特种水产等特色优势产业发展水平。全面加强农产品质量安全管理。加快湖州丝绸小镇、南浔善琏湖笔小镇建设，努力振兴历史经典产业。

深入实施创新驱动发展战略。加大科技投入力度，全社会研究与试验发展经费支出相当于地区生产总值的比例达到2.6%。整合提升科技创新平台，推进国家级莫干山高新区、湖州农业科技园区以及省级现代物流装备高新区等平台建设，积极推动科技城建设，扶持发展一批众创空间。梯度培育科技型企业，大力扶持“高技术、高成长”企业，推进企业研发机构、院士专家工作站建设。全面开展国家科技创新服务体系建设试点，深化政产学研金合作，加快科技大市场建设，促进科技和金融结合，加强知识产权保护和利用。深入实施“南太湖精英计划”等重大人才引育工程。

（四）加快推动新型城市化进程

提升城市发展能级。力争开工建设湖苏沪铁路，全面推进京杭运河湖州段航道整治、长湖申航道西延项目建设，基本完成杭长高速公路北延、 318国道南浔至吴兴段改建工程。整体规划建设长田漾、弁山、图影区域，高质量推进南太湖一体化发展。加强南郊风景区保护和利用。建成奥体中心，启用市民服务中心，完成小西街历史文化街区修缮整治等项目。推进城市地下综合管廊和海绵城市建设。促进各类开发区、产业园区与中心城市、县城、小城市、中心镇融合发展。深入推进“三改一拆”和“无违建”创建工作，深化城市交通拥堵治理，拓展“数字城管”覆盖区域和功能，努力提升城市管理水平。

深化美丽乡村建设。加强村庄规划设计，统筹推进美丽宜居示范村创建和传统村落、历史文化村落保护利用，高标准建设美丽乡村示范带、示范网。在东部平原地区实施城乡环境大治理、大提升，全面推进城乡垃圾减量化、资源化处理，建立健全集镇、村庄常态保洁制度。加大农村交通基础设施建设投入力度。完成新一轮城镇和农村电网改造升级。深化与浙江大学合作共建美丽乡村示范市工作。大力培育现代新型职业农民。深入实施“五年强村计划”，全面帮扶集体经济欠发达村加快发展。

加快构建城乡一体化发展的体制机制。深化农村“三权到人（户）、权跟人（户）走”改革，基本完成确权登记颁证，推动“三权”在不同主体之间的优化配置，鼓励农业转移人口到城镇落户。深化德清县农村集体经营性建设用地入市改革。创新农业经营方式，推进生产合作、供销合作、信用合作“三位一体”的农民合作组织体系建设。扎实开展全国中小城市综合改革试点，加快推进德清国家新型城镇化综合试点。促进“多规合一”，推动全域规划向乡镇覆盖。

（五）努力提升生态文明先行示范区建设水平

大力发展生态经济。全面推进国家级旅游业改革创新先行区、国家全域旅游示范区建设，进一步打响“乡村旅游第一市、滨湖度假首选地”品牌。推动安吉灵峰旅游度假区创建国家级和德清莫干山、南浔古镇创建省级旅游度假区，加快湖州旅游公共服务（集散）中心建设。推进休闲农业与民宿经济等融合发展，培育发展健康养生产业。打造彩色健康森林，发展新型林业经济。大力发展低碳循环经济和节能环保产业。

深入打好水气矿联治攻坚战。深化工业、农业、生活三大污染源治理，提高污水处理设施运行负荷率和达标率。加强河道清淤轮疏和长效保洁管理，开展生态河道建设，努力实现“清三河”达标县区全覆盖。启动太湖治理三大后续工程前期工作。开展圩区整治等新一轮农田水利建设。实行最严格的水资源管理制度，推进水生态文明试点城市建设。深入实施大气污染防治计划，深化污染成因分析，突出废烟、尾气、扬尘等治理重点，强化区域联防联控、多污染物协同控制，加大环境执法力度，确保PM2.5浓度持续下降。发展生态公交，倡导绿色出行。深入推进国家绿色矿业发展示范区建设，继续减点控量，加快废弃矿山生态修复。

全面深化生态文明制度创新。实施《生态文明先行示范区建设条例》，推动全社会生产生活方式绿色化。全面实施环境功能区划，完成永久基本农田划定工作，加强湿地资源保护和利用。扎实推进国家生态文明标准化示范区建设，稳妥推进自然资源资产负债表编制和领导干部自然资源资产离任审计国家试点。支持安吉建设全省“绿水青山就是金山银山”理论实践示范县。深化资源要素配置改革，落实差别价格，推动流转交易，促进土地、能源、环境等资源要素集约高效利用。争创全国绿色金融改革创新综合试验区，探索生态环境资产证券化机制。

（六）不断强化民生改善和社会建设

促进创业就业，强化社会保障。扶持以高校毕业生为重点的青年群体创业，帮扶城镇就业困难人员、农村转移劳动力就业，加强和谐劳动关系建设，城镇新增就业4.8万人，登记失业率控制在4%以内。提升养老、医疗等社会保障城乡统筹发展水平。推进城市棚户区和城乡危旧房改造，进一步完善住房保障体系。健全社会救助体系，整合救助资源，加强兜底保障，大力发展慈善、红十字事业。加快发展养老服务业。推行适度普惠型儿童福利制度。

发展社会事业，优化公共服务。全面深化课程改革，深入开展适应高考招生制度改革的研究和实践，提高各类教育办学质量。稳步实施中小学教育资源布局优化调整，加快智慧校园建设。推进职业教育、特殊教育、民办教育健康发展，加快高等教育内涵、特色发展。推动省教育现代化县区创建工作。深化公立医院综合改革，放开放活民营医院发展。深入实施“双下沉、两提升”工程，完善责任医生签约服务，加快建立分级诊疗制度。优化调整医疗卫生机构布局，加快建设浙北医学中心。确保通过国家卫生城市复评。全面实施一对夫妇可生育两个孩子政策。开展优秀文化弘扬行动，培育和倡导最美风尚，全力争创全国文明城市。加快构建现代公共文化服务体系，推进湖州美术馆等文化设施建设。深化国家历史文化名城保护，推进江南水乡古镇联合申遗工作。促进档案、地方志等事业创新发展。全面筹备第十六届省运会，办好第三届市民运动会。

推进法治建设，创新社会治理。全面启动“七五”普法，加强公共法律服务和法律援助。深化“保平安”专项行动，完善立体化社会治安防控体系，依法严密防范和严厉打击各类违法犯罪活动，确保圆满完成二十国集团领导人峰会等维稳安保任务。推进信访制度、行政复议体制改革，推动人民调解与司法调解、行政调解、民商事仲裁对接联动，着力构建矛盾纠纷多元化解机制。增强城乡社区服务功能，激发社会组织发展活力，推动政府治理、社会调节、居民自治良性互动。加强工矿企业、食品药品等领域事故防范体系建设，提高应急处置能力，确保公共安全形势持续向好。推进军民深度融合发展，深化全民国防教育，加强国防动员和人民防空建设。支持工会、共青团、妇联等人民团体广泛参与社会管理和公共服务，扎实做好民族、宗教、侨务、对台等工作。

各位代表！让全体市民共享改革发展成果，是政府一切工作的出发点和落脚点。今年，我们要全力办好以下十方面民生实事项目：一是大气治理。全面开展城市扬尘治理，创建绿色施工工地40家。开展重点工业企业废气污染整治，完成60家企业挥发性有机废气治理任务。淘汰高污染燃料小锅炉800台。在产矿山全部达到市级以上绿色矿山标准。全面禁止秸秆露天焚烧，秸秆综合利用率达到93%以上。县级以上城区新增有证照餐饮企业油烟净化装置安装率达到100%。二是城乡治水。完成162个行政村的生活污水治理，新增受益农户43895户，农村生活污水治理设施建设基本完成。完成100公里城镇污水配套管网建设。太湖水厂及城乡供水一体化工程一级供水管网建成通水。加快推进太湖流域水环境综合整治四大骨干水利工程建设，完成河道整治68公里、堤防建设120公里。完成中小河道清淤等综合整治270公里。加快河道水质改善，县控以上劣V类、V类水质断面彻底消除，Ⅲ类以上水质占比超过90%。三是食品安全。创建放心农贸市场19家。食品安全信息平台建成并投入使用，优化食品安全检测、信用、巡查、应急等信息的录入和分析处理流程，推进食品安全实时监管。加强对餐饮单位食品安全的监管，新建成“阳光厨房”100家。四是居住环境。新竣工各类保障性住房1万套。创建市级美丽乡村30个。完成540户农村困难群众危旧房改造。开通中心城区小市内河（骆驼桥至衣裳街）、项王公园至太湖、环城河至东龙溪港3条水上旅游观光巴士线路，建成沿线7个专用码头。五是交通建设。新增、更新公交车辆100辆，其中新能源、清洁能源车辆75辆；新增、优化公交线路45条；新建港湾式停靠站120个。新建成农村联网公路80公里，完成农村公路等级提升180公里。加快推进新能源汽车充电设施建设，市区建成快速充电站8座、公共充电桩200个。加快建设商合杭铁路湖州段、申嘉湖高速公路西延工程，开工建设杭宁高速公路改扩建工程。中心城区仁皇山大桥项目竣工投入使用，湖山大道项目（三环北路至滨湖大道）全面启动。六是敬老养老。启动市老年大学扩建项目。新建成城乡社区居家养老服务照料中心200个。为1.7万名困难失能老人实施政府购买居家养老服务。七是医疗服务。组织二级以上公立医院开展200场“进村入企”医疗卫生服务。完成红十字应急救护普及培训5.5万人以上。八是教育文体。新建成农村文化礼堂80个。开展全民健身公益大培训，培训不少于8万人次。推动优质教育资源均衡化，新建成中小学校录播教室50个，实现录播教室乡镇全覆盖。加快特殊教育发展，残疾儿童幼儿园入园率达到80%以上，适龄“三残”儿童少年义务教育入学率达到98%。九是就业帮扶。帮扶困难人员和残疾人创业就业分别达到4000人、3000人。完成对大学生、农村青年等人员创业培训3000人，完成高技能人才培训1万人。十是公共服务。开工建设市城市规划展览馆。在城乡社区新建成“E邮柜”等电子商务投递终端100个，改造提升农村电商服务站200家。城乡居住小区周边新增视频摄像点3000个，实现公共区域视频监控全覆盖。完成市殡仪馆改扩建一期工程并投入使用。

四、切实加强政府自身建设

新常态要有新状态。着力新一年，干好“十三五”，必须坚持以政府改革创新引领全社会创业创新。我们要以更高的标准、更严的要求，切实加强政府自身建设，努力为经济社会发展打造符合法治精神、充满生机活力、更加廉洁高效的政务生态系统。（一）狠抓执行落实，着力打造担当型政府。深入践行“三严三实”，增加政府履行职责的约束性指标，使大抓落实、马上就办成为广大政府工作人员的行动自觉。以“钉钉子”的精神坚决贯彻中央和省、市委各项决策部署，确保政令畅通、步调一致。以当前政府工作中存在的短板为突破口，认认真真找准短板，扎扎实实补好短板。紧盯“十三五”规划落地，建立“六重”事项动态完善、年度推进、责任分解、考核评估工作机制，一桩桩、一件件抓好落实。进一步精简各类文件、会议、活动，腾出更多精力服务基层、推动发展。强化督查问责、跟踪问效，大力整肃行政不作为、慢作为、乱作为，不断提升政府执行力和公信力。

（二）突出治理创新，着力打造服务型政府。更多运用市场化、社会化、信息化方式，创新公共服务供给，提升公共服务效率。积极探索推进政府向社会组织转移职能、向社会力量购买服务。进一步提升“12345政府阳光热线”功能，完善服务群众快速响应机制。加强乡镇（街道）社会综治、市场监管、综合执法、便民服务等平台建设，着力构建统一高效的基层治理体系。实施“互联网＋”提升政府治理能力工程，推动建立与现代治理相适应的智慧政务体系。

（三）加强依法行政，着力打造法治政府。政府一切活动都要全面纳入法治轨道，全体公务人员要带头学法守法、依法办事，自觉运用法治思维和法治方式深化改革、推动发展、化解矛盾、维护稳定。加快构建现代政府职责体系，所有行政行为都要于法有据，任何部门都不得法外设权。配合人大做好重点领域立法工作，全面落实行政规范性文件和行政合同备案监督管理制度。健全行政决策机制，加强新型智库建设，进一步完善决策咨询、法律顾问等制度。深化阳光行政，推进政府信息公开。

（四）全面从严治党，着力打造廉洁政府。把纪律和规矩挺在前面，严格落实政府系统党风廉政建设主体责任，以零容忍的态度坚决惩治腐败。认真贯彻中央“八项规定”精神和国务院“约法三章”，推进作风建设常态化、长效化。自觉接受人大监督、民主监督、司法监督、社会监督、舆论监督，支持纪检监察机关监督执纪问责，提升审计监督效力。健全公共资源交易、政府投资管理、公共基金监管、国有资本运作等制度体系，扎紧制度“笼子”，规范权力运行。

各位代表！发展成就鼓舞人心，发展前景催人奋进。湖州正站在赶超发展的新起点上。让我们在中共湖州市委的领导下，凝聚全市人民的智慧和力量，坚定信心、振奋精神，勇于担当、开拓进取，努力实现“十三五”良好开局，为加快建设现代化生态型滨湖大城市，高水平全面建成小康社会而努力奋斗！
